# *MYCN* Amplification Is Associated with Repressed Cellular Immunity in Neuroblastoma: An *In Silico* Immunological Analysis of TARGET Database

**DOI:** 10.3389/fimmu.2017.01473

**Published:** 2017-11-03

**Authors:** Peng Zhang, Xiaofang Wu, Moushumi Basu, Chen Dong, Pan Zheng, Yang Liu, Anthony David Sandler

**Affiliations:** ^1^Center for Cancer and Immunology Research, Children’s National Health System, Washington, DC, United States; ^2^Sheikh-Zayed Institute for Pediatric Surgical Innovation, Children’s National Health System, Washington, DC, United States

**Keywords:** *MYCN*, immunesuppression, neuroblastoma, cellular immunity, *in silico* analysis

## Abstract

**Purpose:**

RNA and DNA sequencing data are traditionally used to discern intrinsic cellular pathways in cancer pathogenesis, their utility for investigating the tumor microenvironment (TME) has not been fully explored. This study explores the use of sequencing data to investigate immunity within the TME.

**Experimental design:**

Here, we use immune cell fraction estimation analysis to determine the immune profiles in the microenvironment of neuroblastoma (NB) based on RNA-seq data in the TARGET database. The correlation between immune cell transcripts and prognosis in pediatric NB is also investigated.

**Results:**

*In silico* analysis revealed a strong inverse correlation between *MYCN* amplification and leukocyte infiltration. This finding was validated by immunohistochemistry analysis in tumor samples. Moreover, the abundance of CD4 T cells strongly associated with better patient survival regardless of *MYCN* gene amplification, while those of CD8 T cells, NK or B cells do not. Based on characteristic cytokine expression of CD4 subsets in tumors, the Th2 rather than Th1 levels were associated with better prognosis.

**Conclusion:**

We found that the *in silico* analysis of TARGET database reflected tumor immunity and was validated by the immunohistochemical tumor data. Our results reveal the association of *MYCN* amplification with repressed cellular immunity and the potential prognostic value of infiltrating CD4 T cell transcripts in pediatric NB. This analysis illustrates the potential role of *MYCN* in NB as a regulator of immune privilege and characterizes the power of *in silico* analysis for delineating cancer immunology and risk stratification.

## Introduction

Neuroblastoma (NB) is among the most common cancers in childhood. More than 90% of NB are diagnosed prior to 5 years of age, in which the prognosis of NB varies greatly based on risk stratification (1–3). While >90% of patients with intermediate and low-risk tumors survive 5 years or more, only 40–50% of patients with high-risk NB achieve 5-year survival (4). Risk stratification is based on genetic alterations, histological findings as well as age of diagnosis among other clinical factors. While *MYCN* amplification and unbalanced 11q aberration have emerged as the dominant factors in risk assignment (5), poorly differentiated or undifferentiated ganglioneuroblastoma, and NB without these genetic alterations can also show poor prognosis. It is therefore of interest to search for other shared features of high-risk NB identified by either histology or genetic risk profiles.

The theory of immune surveillance, as originally proposed by Burnet, predicts that immune response is a key determinant of cancer risk (6). In a 1972 study of histologic lymphocytic infiltration in 23 primary NBs, a positive correlation with survival was found in infancy and childhood (7). Through extensive investigations, it is established in a number of other tumor types that components of immune infiltration into the tumor plays a critical role in cancer prognosis. However, these analyses are difficult to standardize, thus it has not been feasible to use them as markers for risk assignment in cancer prognosis.

Whole genome sequencing of tumor samples is now routinely performed for cancer samples. The wealth of data collected from these studies allows one to unveil molecular pathogenesis of cancer cells. Since host inflammatory cells are now considered an integral part of the tumor microenvironment (TME), it is inevitable that deep sequencing of cancer tissues will also capture information of inflammatory cells. CIBERSORT is a recently reported computational approach that uses gene expression profiles to estimate relative fractions of diverse cell subsets in complex tissues, including tumors (8, 9). The power of this algorithm in delineating immune composition of tumor tissues prompted us to test whether one can use the bulk of next generation sequencing data already collected to evaluate the potential role of immunity in determining prognosis in NB, a tumor with well-defined risk stratification. Here, we report our use of this program to analyze TARGET data matrix for NB in which we found that CD4 T cell signature significantly associates with prognosis of NB in both *MYCN* amplified and non-amplified tumors. We also observed the unexpected association between *MYCN* gene amplification and lack of inflammatory profiles suggesting the impact of *MYCN* gene amplification in repressing NB host immunity. These findings were verified by selective immunohistochemistry in patient samples. Furthermore, these data show the power of *in silico* analysis for cancer immunology and raises the intriguing possibility of assigning cancer risk based on nextgen RNA-seq data.

## Materials and Methods

### Data Source

The TARGET initiative provides sequencing data and de-identified clinical information of cancer patients (available through the TARGET Data Matrix^1^). The current studies utilized database of genotypes and phenotypes *via* accession phs000471. A total of 148 NB patients were included based on availability detailed RNA-seq FPKM data, survival time after diagnosis, risk assignment, and *MYCN*-amplification status. The demography, risk stratification of NB with or without *MYCN* amplification were summarized in Table 1.

**Table 1:** Baseline characteristics of the patients.*

Characteristic	Patients with *MYCN* amplification (*n* = 31)	Patients without *MYCN* amplification (*n* = 117)	Statistical significance (*P-*value)
**Age at diagnosis—days**			
Mean ± SEM	1,037.52 ± 162.672	1,303.18 ± 101.853	ns (0.22)
**Sex—no. (%)**			
Male	18 (58.1)	69 (59.0)	ns (>0.99)
Female	13 (41.9)	48 (41.0)	ns (>0.99)
**COG risk group—no. (%)**			
High risk	31 (100.0)	91 (77.8)	Significant (0.0024)
Intermediate risk	0 (0.0)	13 (11.1)	ns (0.07)
Low risk	0 (0.0)	13 (11.1)	ns (0.07)

*P-values were calculated by using two-tailed Student’s *t*-test (age at diagnosis) and Fisher’s exact test [sex and Children’s Oncology Group (COG) risk Group].

### Immune Cell Fraction Estimation Analysis

The online analytic tool, CIBERSORT (Cell type Identification By Estimating Relative Subsets Of known RNA Transcripts), was used according to the instruction provided by the developers.^2^ LM22, a validated leukocyte gene signature matrix, was used here as a gene signature matrix. LM22 contains 547 genes that distinguish 22 human hematopoietic cell phenotypes. The gene expression data from the NB patients in TARGET database (*n* = 148) were input as a Mixture file and 500 permutations were performed. The percentage data of 22 immune cell types in each patient were transferred into the cell fraction of tumor by normalization with the ratio of *CD45* and *ACTB* expression value. Additionally, 22 human hematopoietic cell phenotypes were merged into 16 immune cell types as follows: naive and memory B cells were merged into B cells; naïve, memory, resting, and activated CD4 T cells were merged into CD4 T cells; NK cells resting and NK cells activated were merged into NK cells; dendritic cells resting and dendritic cells activated were merged into DC cells; while mast cells resting and mast cell activated were merged into mast cells.

### Stratification of Subgroups Based on Expression and Fraction Data

For CD45 expression, the NB samples were divided into *CD45^hi^* and *CD45^lo^* groups with the threshold (FPKM = 7.5) based on its bimodal distribution of RNA-seq data (Figure S1A in Supplementary Material). Gene expression levels were normalized based on actin expression and samples were divided into higher ratio (above the upper quartile) and lower ratio (below the lower quartile) groups based on RNA-seq data. For other parameters, the samples were divided into lower cell fraction (below the fraction value of peak) and higher cell fraction (above the twofold of fraction value of peak) groups based on their data distribution of estimated cell fraction (T cells CD4 cell fraction distribution shown in Figure S1B in Supplementary Material).

### Gene Set Enrichment Analysis (GSEA)

Gene set enrichment analysis version 2.3.0^3^ was performed with the JavaGSEA application using the following parameters: number of permutations = 1,000, permutations of phenotype, curated KEGG gene sets from MSigDB as gene sets database input. Significant enrichment was defined as those lists with >30 genes and a false discovery rate <0.05.

### Specimens

Tumor specimens were obtained from 15 patients diagnosed with low (*n* = 3), intermediate (*n* = 6), and high (*n* = 6) risk NB. Diagnosis and staging were performed according to Children’s Oncology Group protocols. Biopsies were taken at the time of diagnosis and prior to initiation of any therapy. Specimen collection was obtained with the appropriate research consent (and assents when applicable) and was approved by the Institutional Review Board, CNMC, Washington D.C. (Pro00004284).

### Immunohistochemical Analysis

Five-micron paraffin tissue sections were deparaffinized in xylene and rehydrated in 100 and 95% ethanol. Endogenous peroxidase activity was blocked by treating with 0.3% hydrogen peroxide for 30 min followed by phosphate-buffered saline (PBS) washing for 5 min. Sections were incubated overnight at 4°C with primary antibodies diluted in blocking buffer (PBS with 5% serum). The following primary antibodies were used: CD4 (rabbit monoclonal antihuman IgG, 1:150, abcam, Cambridge, MA, USA); CD45 (mouse monoclonal antihuman IgG1, abcam). Isotype-matched antibodies were used for negative controls. After washing with PBS, each section was incubated with 1:200 diluted biotinylated secondary antibodies (Vector Laboratories, Inc., Burlingame, CA, USA) for 1 h at room temperature. Secondary antibody was detected with Vectastain ABC reagent (ABC Elite Kit; Vector Laboratories), and 3′3′diaminobenzidine substrate (Sigma). Slides were washed in tap water for 5 min and then counterstained with hematoxylin (Sigma) for 30 s. Following counterstaining, slides were washed in tap water for 5 min, dehydrated (95 and 100% ethanol), transferred to 100% xylene, and mounted. Brightfield immunohistochemical images were acquired with a Nikon Eclipse E800 microscope (Nikon Corp.). Images were taken at magnifications of ×100.

### Quantitative Analysis of CD4 and CD45 Expression in Human Tissue

Thirty to forty randomly selected fields in each stained specimen were imaged under ×100 magnification. Quantification of positive staining intensity was achieved using Olympus cellSens imaging software (version 1.7). The positive staining intensity and the area of tumor tissue were measured. The positive staining intensity in each field was measured using a manual threshold setting. Measurements were made by the same person who was blinded to the clinical stage or characteristics of the tumor specimen. Data are presented as the mean positive staining intensity of all the fields for each specimen.

### Biostatistics

The specific tests used to analyze each set of experiments are indicated in the figure legends. Data were analyzed using an unpaired Mann–Whitney test to compare between two groups, and χ^2^ test for contingency table. In the graphs, *y*-axis error bars represent SEM as indicated. Statistical calculations were performed using GraphPad Prism software (GraphPad Software, San Diego, CA, USA) or R Software.^4^

## Results

### *MYCN* Amplification Is Associated with Reduced Inflammation in NB

**Figure 1:**
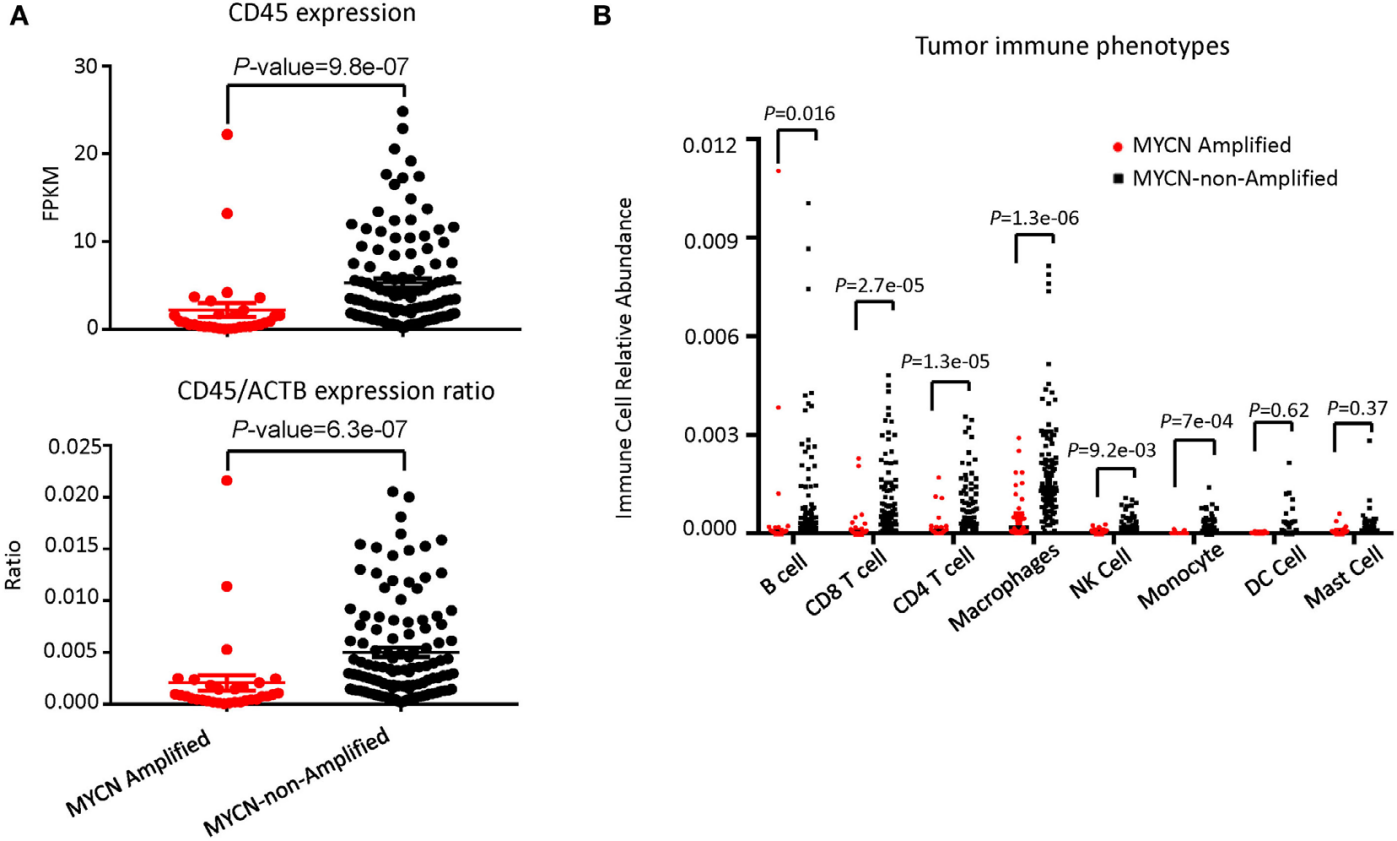
*MYCN* amplification in neuroblastoma (NB) affects inflammatory immune cell phenotypes. **(A)** *MYCN*-non-amplified NB samples had greater inflammatory status as defined by CD45 expression (upper panel) and CD45/actin expression ratio (lower panel). **(B)** The relative abundance of six immune cell types was greater in *MYCN*-non-amplified tumors than in *MYCN*-amplified tumors. Statistical significance in **(A,B)** was determined using Mann–Whitney test.

To determine whether the increased risk is associated with either reduced immune response or increased resistance to it, we tested whether the inflammatory response is associated with *MYCN* amplification among patients in the TARGET database. The patients with or without *MYCN* amplification are similar in age at diagnosis, gender distribution (Table 1). We chose the transcript levels of the CD45 genes that is the marker for all leukocytes, among *MYCN* amplified or non-amplified NB tumors. As shown in Figure 1A, after normalizing against *ACTINB* transcripts, *MYCN* amplified NB had significantly lower levels of CD45 transcripts, indicating reduced inflammation in *MYCN* amplified NB. To determine whether *MYCN* affects specific subset of leukocytes, we used the CIBERSORT algorithm to estimate the percentage of each leukocyte subset, focusing on the major cell types that can be identified by CIBERSORT. Among the major immune effector cells, including T, B, macrophages, dendritic cells, and NK cells, we found a general reduction of all leukocytes in *MYCN*-amplified samples (Figure 1B). The strong inverse correlation suggests that *MYCN* amplification in cancer cells has a profound impact on host immune response to NB.

**Figure 2:**
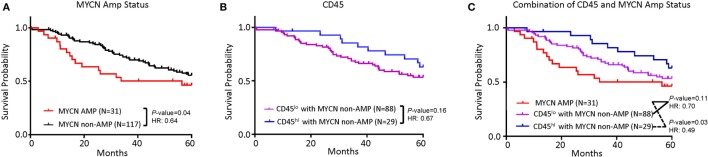
Tumor inflammation, combined with *MYCN* amplification is associated with neuroblastoma (NB) prognosis. **(A)** Kaplan–Meier curves showing 5-year survival of NB patients associated with *MYCN* amplification status. **(B)** Kaplan–Meier curves showing 5-year survival of NB patients assessed by *CD45* expression. *CD45^lo^* and *CD45^hi^* subgroups were defined based on FPKM distribution as detailed in Section “Materials and Methods.” **(C)** Kaplan–Meier curves showing 5-year survival of NB patients according to the combination of *MYCN* amplification status and CD45 expression subtypes. The hazard ratio and *P*-value in **(A,B,C)** displayed on the graphs are from the log-rank test and Gehan–Breslow–Wilcoxon method.

While poor prognosis *MYCN*-amplified NB is well accepted and clearly demonstrable in the TARGET sample cohort (Figure 2A), the risk of *MYCN* non-amplified samples is traditionally stratified by a comprehensive list of clinical, pathological, and molecular criteria. To determine if the abundance of CD45 transcripts can be used as a single parameter for NB risk stratification in *MYCN* non-amplified NB, we evaluated if CD45 levels associate with risk in this subgroup. As shown in Figure 2B, samples with more abundant *CD45* show better survival, but the difference between *CD45^hi^* and *CD45^lo^* samples was not statistically significant. The hazard ratio (HR) and separation of the two groups suggest that lack of statistical significance may be due to a limited number of *CD45^hi^* samples as the TARGET samples are biased toward high-risk group stratification. Combining *MYCN* non-amplification and *CD45^hi^* parameters identified a group of NB patients with better survival (HR = 0.49, *P* = 0.03) (Figure 2C).

### Abundance of CD4 T Cells in NB as a Biomarker for Overall Survival

**Figure 3:**
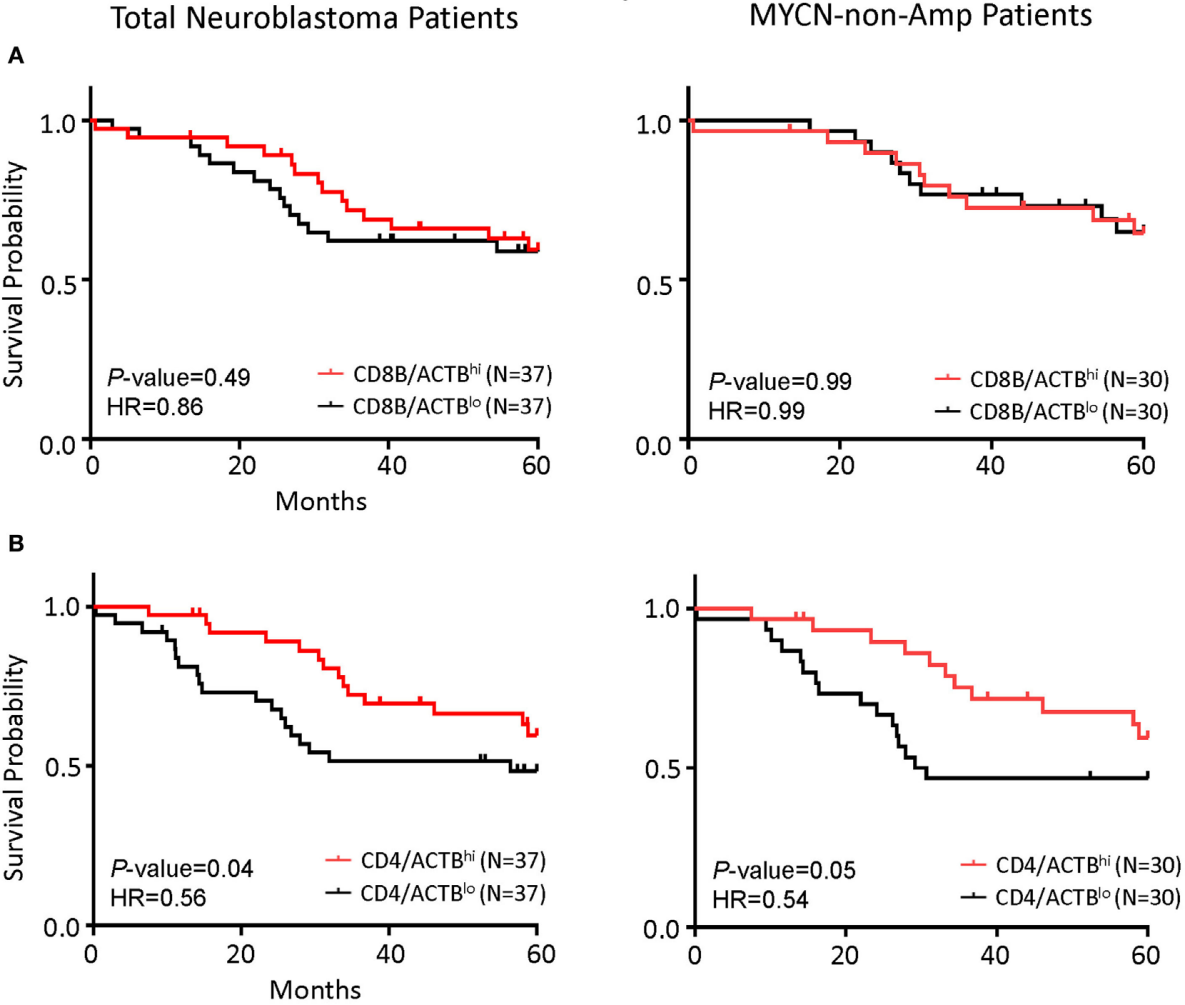
*CD4* gene expression in the tumor is the principal determinant of prognosis in all patients and in patients with *MYCN* non-amplified tumors. **(A)** Kaplan–Meier curves showing 5-year survival of neuroblastoma (NB) patients according to their *CD4* gene expression normalized to the ratio of actin expression. Total NB samples (left) and *MYCN* non-amplified NB samples (right) were divided into *CD4/ACTB^hi^* (above the upper quartile) and *CD4/ACTB^lo^* (below the lower quartile) groups based on RNA-seq data. **(B)** Kaplan–Meier curves showing 5-year survival of NB patients according to their CD8B gene expression normalized with the ratio of actin expression. Total NB samples (left) and *MYCN* non-amplified NB samples (right) were divided into *CD8B/ACTB^hi^* (above the upper quartile) and *CD8B/ACTB^lo^* (below the lower quartile) groups based on RNA-seq data. The hazard ratio and *P*-value in **(A,B)** displayed on the graphs are from the log-rank test and Gehan–Breslow–Wilcoxon method.

As the first test to determine whether T cells play a significant role in NB prognosis, we compared the overall survival of the NB patients with the highest 25% or the lowest 25% levels of the gene transcript. While the *CD8B^hi^* and *CD8B^lo^* NB patients show nearly identical overall survival (Figure 3A), the *CD4^hi^* NB patients have significantly better survival (Figure 3B).

**Figure 4:**
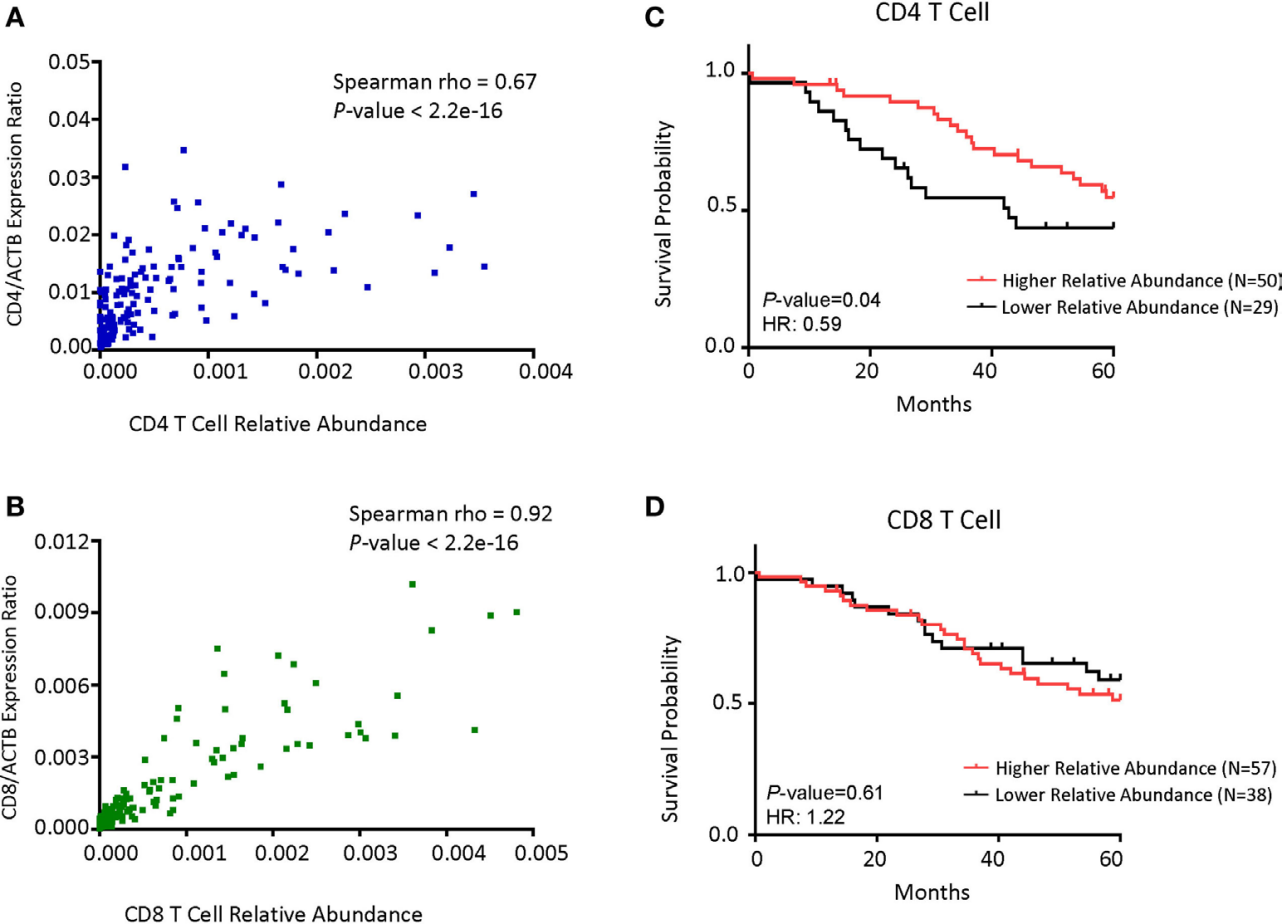
CD4 cell abundance is the principal determinant for prognosis in patients with *MYCN* non-amplified tumors. **(A)** Dot plot showing the correlations between *CD4* gene expression normalized with the ratio of actin expression and CD4 T cell relative abundance. **(B)** Dot plot showing the correlations between *CD8B* gene expression normalized with the ratio of actin expression and CD8 T cell relative abundance. **(C)** Kaplan–Meier curves showing 5-year survival of neuroblastoma (NB) patients with *MYCN* non-amplified tumors according to their CD4 T cell relative abundance. **(D)** Kaplan–Meier curves showing 5-year survival of NB patients with *MYCN* non-amplified status according to their CD8 T cell relative abundance. NB samples were divided into Relative Abundance^hi^ and Relative Abundance^lo^ groups, respectively, based on the immune cell relative abundance data as detailed in Section “Materials and Methods.”. Correlation coefficient and *P*-value in **(A,B)** were determined using Spearman method. The hazard ratio and *P*-value displayed on the graphs are from the log-rank test and Gehan–Breslow–Wilcoxon method.

To confirm whether the abundance of *CD4* or *CD8B cell* transcripts can be used as surrogate markers for the abundance of T cell subsets, we performed regression analysis of the transcript levels for the CD4 and CD8 T cells. As shown in Figure 4A, the levels of *CD4* transcripts strongly correlated with the fraction of CD4 T cells among all leukocytes. Likewise, the levels of *CD8* transcripts strongly associates with the CD8 cell fraction (Figure 4B). To determine whether a specific leukocyte population may be associated with overall survival, we divided the samples into high- and low-populations based on the relationship with peak values. Those below peak value are assigned as the low subsets, while those that are 3-fold of the peak value are defined as the high subset. As shown in Figure 4C, patients with the CD4 T cells^hi^ subsets in the tumor have much better overall survival than those with the CD4 T cells^lo^ subset. In contrast, patients with the CD8 T cells^hi^ subset had similar overall survival to that of CD8 T cells^lo^ subset (Figure 4D).

**Figure 5:**
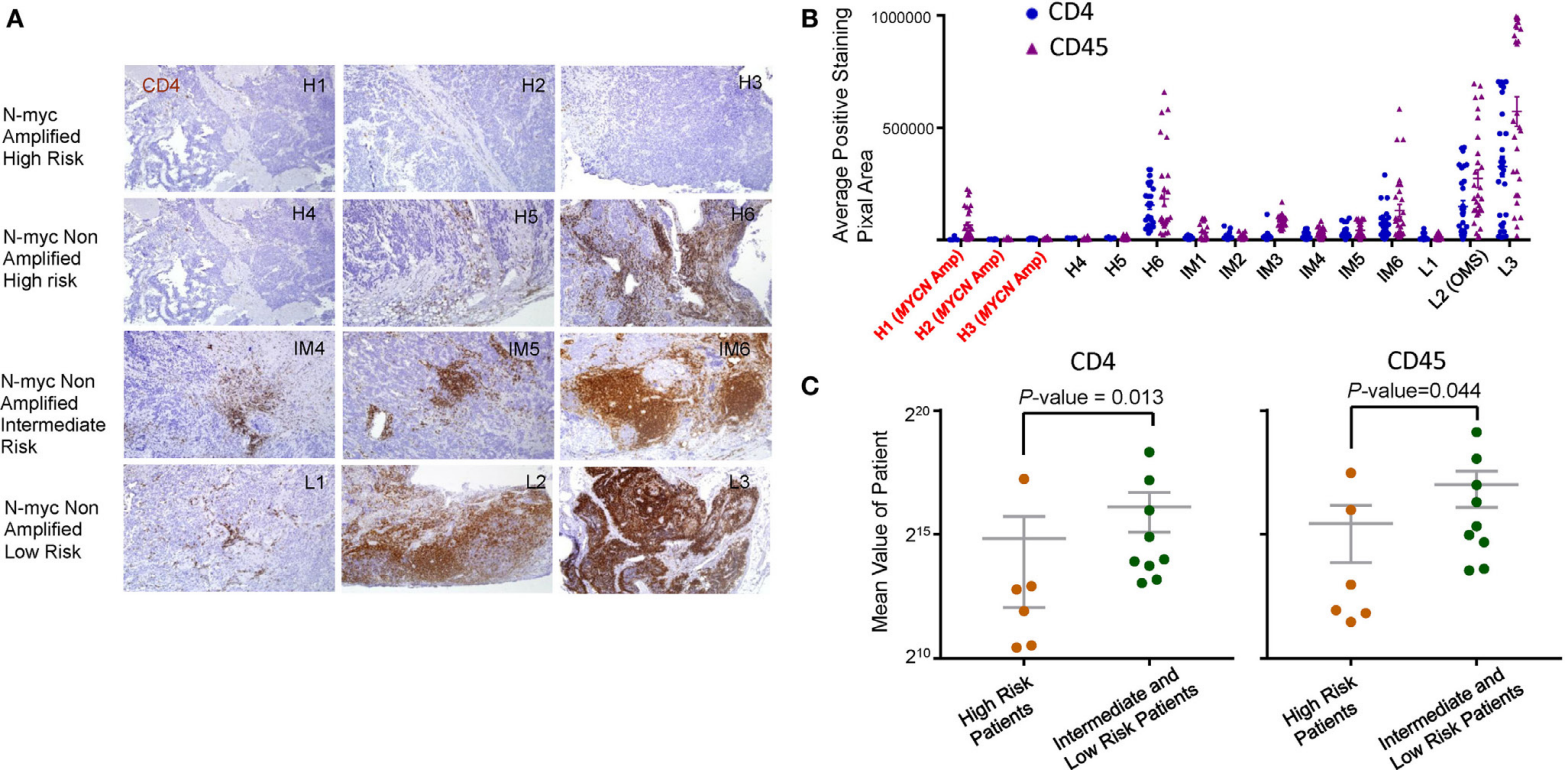
Immunohistochemical analysis of leukocyte infiltration in neuroblastoma (NB). **(A)** Immunohistochemical staining of CD4 (brown) and CD45 (data not shown) was performed on paraffin embedded NB tumor tissue at diagnostic biopsy from *MYCN* amplified high-risk, *MYCN* non-amplified high-risk, *MYCN* non-amplified intermediate, and *MYCN* non-amplified low-risk tumors. The nuclei were counterstained with hematoxylin (blue). Tissue sections were imaged at ×100 original magnification. The association between CD4, CD45 expression, and *MYCN* amplification **(B)** and risk stratification **(C)** was determined by digital mean density image analysis and the data are graphed. *P*-values in **(C)** were calculated with an unpaired Mann–Whitney test.

We performed selective immunohistochemistry analysis of NB samples to confirm the key findings of the above *in silico* analyses. As shown in Figures 5A,B, all *MYCN*-amplified samples analyzed had minimal infiltration of CD4 T cells. While high-risk *MYCN* non-amplified NB are similar in CD4 T cell infiltration, intermediate, and low-risk NB had variable amounts of CD4 T cells infiltrates. As expected, the pattern of CD45 staining is similar to the CD4 T cells (data not shown). When the staining of CD45 and CD4 was integrated digitally, it is clear that high-risk NB samples (including *MYCN* amplified and non-amplified) had reduced CD4 and CD45 staining (Figure 5C). These data validated the *in silico* analysis.

### Immunological Mechanism of Protection by CD4 T Cells

**Figure 6:**
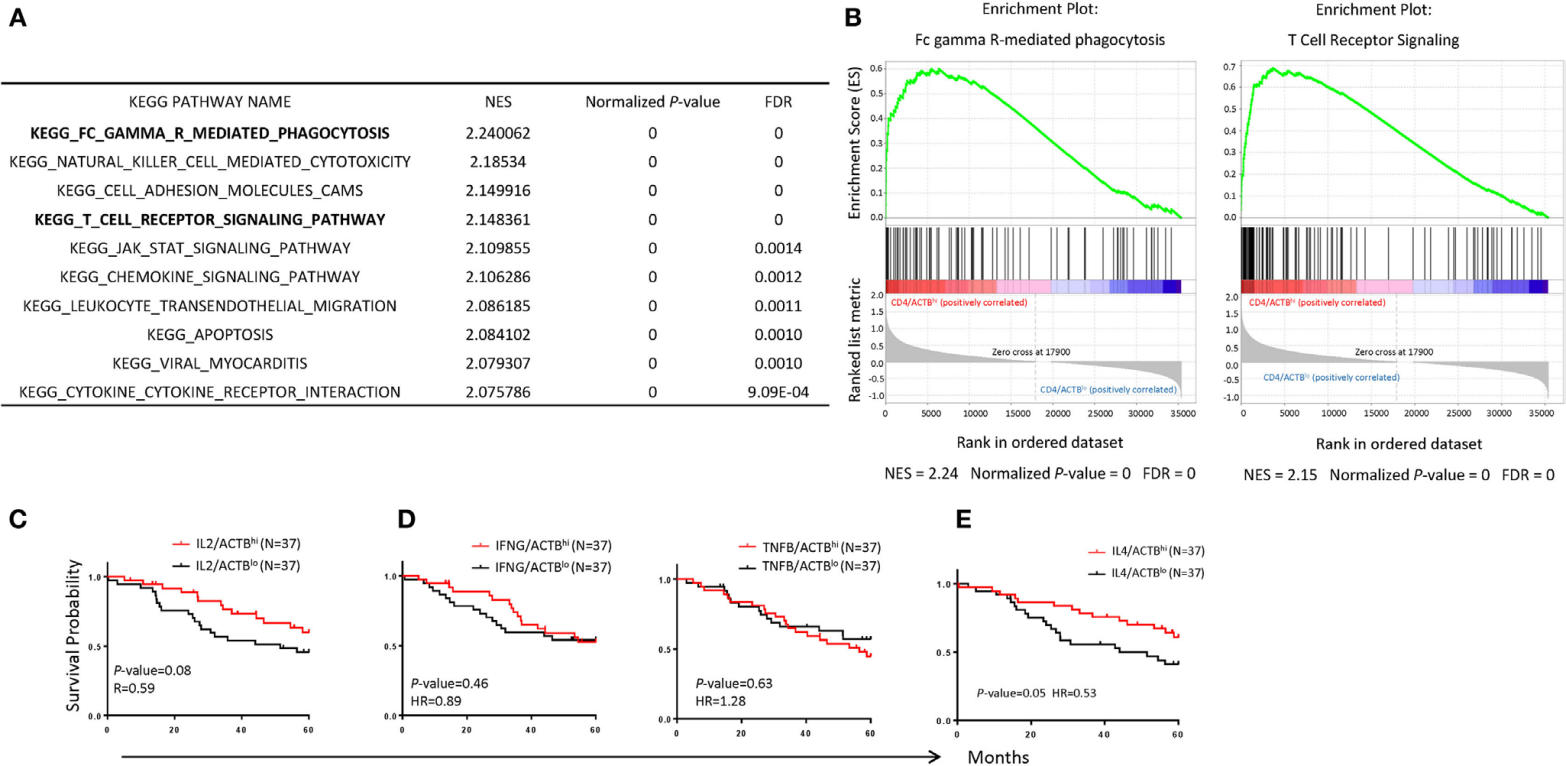
Immunological mechanisms underlying the association between CD4 T cells and prognosis of neuroblastoma (NB) patients. **(A)** Top 10 significant KEGG pathways enriched in NB samples with *CD4^hi^*. The normalized enrichment score normalized *P*-value and false discovery rate are displayed on the graphs. **(B)** Gene set enrichment analysis enrichment plot showing significant enrichment of upregulated pathways (Fc gamma R-mediated phagocytosis; left, T cell receptor; right) in NB samples with *CD4^hi^*. **(C–E)** Kaplan–Meier curves showing 5-year survival of NB patients according to their *IL2* **(C)**, *IFNG*, and *TNFB* **(D)**, and *IL4* **(E)** gene expression normalized with the ratio of actin expression. The NB samples were divided into high- and low-groups based on their upper and lower quartile of RNA-seq data. The hazard ratio and *P*-value displayed on the graphs are from the log-rank test and Gehan–Breslow–Wilcoxon method.

To understand the possible mechanism by which increased CD4 T cells confer protection for NB patients, we performed Kegg pathway analysis to identify enhanced pathway in the samples in high-CD4 transcripts. The top 10 pathways are listed in Figure 6A and examples of the association are shown in Figure 6B. Chief among them include FcRγ-mediated phagocytosis, NK cell-mediated cytotoxicity, cell adhesion molecules, and T cell signaling. These data suggest that CD4 T cells likely promote cancer rejection by orchestrating innate immune effectors within the TME.

CD4 T cells can be divided into regulatory and effector T cells. Since our preliminary analysis suggest no correlation between Treg subsets (data not shown), we focused on effector CD4 cells. CD4 T cells differentiated into multiple functional subsets including Th0, Th1, Th2, Th9, and Th17 among others, each characterized by their cytokine profiles and their master transcriptional regulators. Since most master regulators are prominently expressed in cancer cells, we decide to use their characteristic cytokines to discern the functional subsets associated with NB prognosis. As shown in Figures 6C–E, IL-2 (Th0), IFNγ, and TNFα (Th1) transcript levels showed no statistically significant association with NB prognosis. Surprisingly, IL-4 transcript levels were significantly associated with NB prognosis (*P* = 0.05).

## Discussion

Neuroblastoma is the most common extra-cranial solid tumor found in children and continues to have a poor prognosis in cases of high-risk disease despite multimodal therapy (10–12). Risk stratification is well defined in NB which predicts patient outcome, but is also used to guide therapy. Risk stratification is based on molecular, pathologic, and clinical findings, but there are no immunologic parameters that are considered in defining tumor risk (3). As immunotherapy in the form of either targeted antibodies or checkpoint inhibitors is changing cancer treatment, understanding the immune environment could be critical to both risk stratification, and application of immunotherapy. Using *in silico* analysis from a sequence database, we investigated NB from the perspective of immune cell transcript infiltrates and the association with outcome. It was clear that CD4 transcripts were associated with better outcomes in which a high transcript was associated with improved survival. This correlated with immunohistochemistry for CD4 cellular infiltrates in which tumors with poor prognosis and high risk had a lower CD4 cellular infiltrate in tumors. Though it is established in a number of tumor types that components of immune infiltration into the tumor plays a critical role in cancer prognosis (13–18), standardizing cellular infiltrates has been difficult and thus application for risk stratification has not been practical. Analyzing transcripts in sequence data may offer a method for standardizing cellular infiltrates for use in risk assignment for cancer prognosis.

*MYCN* amplification in NB is the most important molecular biomarker categorizing tumors in the high-risk category. Irrespective of all other clinical, molecular, or biomarkers, if *MYCN* is amplified in the tumor it is categorized as high risk. In our analysis, *MYCN* amplified NB had significantly lower levels of CD45 transcripts, indicating reduced inflammation in *MYCN* amplified tumors. All the transcripts of major immune effector cells, including T cells, B cells, macrophages, dendritic cells, and NK cells, were significantly reduced in *MYCN*-amplified tumors compared with non-amplified tumors. This observation could have profound implications for the role of *MYCN* in NB biology. *MYCN* drives many of the “hallmark” features of NB that include tumorigenicity, cellular proliferation, and growth as well as protein synthesis and altered cellular metabolism (19, 20). The finding of repressed immune cell infiltrates in *MYCN* amplified tumors implies a critical role for evading immunity and may thus be a potential target for inducing immunogenicity. Indirect evidence is observed in the opsoclonus–myoclonus syndrome associated with low or intermediate stage NB. These tumors have profound T-cell infiltrates and are not *MYCN* amplified (21–23). The OMS phenomenon is thought to be an autoimmune effect and along with NB that spontaneously regresses this tumor may be an ideal model for studying and understanding tumor immunity as it relates to tumor biology and prognosis. Of particular interest, recent findings indicate that immunity plays a key role in tumor regression following oncogene inactivation (24, 25). Experimentally, inactivation of MYC in genetically engineered conditional mouse tumor models, induced tumor regression when host immunity was intact (26). CD4 helper cells were found to be critical to this effect, suggesting that MYC oncogenicity occurs not only through tumor-intrinsic mechanisms but also through host-dependent immune mechanisms. Our findings from patient tumors confirm this phenomenon of *MYCN* amplified tumors having a profound effect on cellular immunity.

In summary, our data show the association of *MYCN* amplification with repressed cellular immunity as well as the association of infiltrating CD4 T cell transcripts with improved prognosis in pediatric NB. These data illustrate the potential role of *MYCN* in NB as a regulator of immune privilege and characterizes the power of *in silico* analysis for understanding cancer immunology and risk stratification.

## Ethics Statement

Specimen collection was obtained after appropriate research consents (and assents when applicable) and was approved by the Institutional Review Board, CNMC, Washington D.C. (Pro00004284). All information obtained was protected and de-identified.

## Author Contributions

PengZ mined data, performed analysis, and manuscript preparation. XW performed experiments and manuscript preparation. MB performed experiments. CD and PanZ reviewed data and manuscript preparation. YL and AS formulated concept, reviewed data, and manuscript preparation.

## Conflict of Interest Statement

The authors declare that the research was conducted in the absence of any commercial or financial relationships that could be construed as a potential conflict of interest. The reviewer MG and handling editor declared their shared affiliation.

## Footnotes

^1^http://target.nci.nih.gov/dataMatrix/TARGET_DataMatrix.html.

^2^https://cibersort.stanford.edu/.

^3^http://www.broadinstitute.org/gsea.

^4^https://www.r-project.org/.
