# Supplementary material for: MYCN Amplification Is Associated with Repressed Cellular Immunity in Neuroblastoma: An In Silico Immunological Analysis of TARGET Database
Source: Front Immunol. 2017 Nov 3;8:1473. doi: 10.3389/fimmu.2017.01473 (PMC5675839; doi:10.3389/fimmu.2017.01473)
Supplement: Supplementary file 1 [file data_sheet_1.docx]

**Supplementary Figure 1. Data distribution of CD45 expression value and CD 4 T cell relative abundance** (a) Density plot shows the FPKM distribution of *CD45*. Neuroblastoma samples were divided into *CD45^hi^* (above 7.5) and *CD45^lo^* (below 7.5) groups based on RNA-seq data. (b) Density plot shows the estimated cell relative abundance distribution of T CD4 cell. Neuroblastoma samples were divided into lower cell relative abundance (below the peak,1.3e-4) and higher cell relative abundance (above the two fold of peak,2.6e-4) groups.
